# Increased Three-Year Mortality Was Observed During COVID-19 Pandemic Among Patients Discharged from the Acute Rehabilitation Ward After Acetabular and Femoral Fracture Surgery

**DOI:** 10.3390/medicina62040650

**Published:** 2026-03-29

**Authors:** Slađana Vuković Baras, Asija Rota Čeprnja, Dinko Pivalica, Renata Kožul Blaževski, Andrija Jukić, Ljupka Barić, Dušanka Martinović Kaliterna, Jure Aljinović

**Affiliations:** 1 Division of Physical Medicine and Rehabilitation with Rheumatology, University Hospital of Split, Šoltanska 1, 21000 Split, Croatia; 2 Faculty of Health Sciences, University of Split, 21000 Split, Croatia; 3 University Department of Professional Studies, University of Split, 21000 Split, Croatia; 4 School of Medicine, University of Split, 21000 Split, Croatia

**Keywords:** comorbidity, complication, COVID-19, fracture, major illness, mortality, rehabilitation

## Abstract

*Background and Objectives:* Hip fracture surgery is considered a major operation due to the risk of complications and increased mortality. COVID-19 is a newly recognized risk factor for increased mortality in regard to various diseases. *Materials and Methods*: The aim of this retrospective observational study, conducted from January 2018 to April 2022, was to analyze mortality among rehabilitation ward patients after surgical treatment of acetabular or femoral fractures in both the COVID-19 and pre-COVID-19 periods. The association between mortality and age, gender, comorbidity status, and number of complications during hospital stay was also examined. *Results*: Higher mortality was observed in the COVID-19-period group during all analyzed periods: cumulative three-year mortality was 2.3 times higher (14.2% vs. 6.2%, *p* = 0.013); two-year mortality was 3.7 times higher (9.2% vs. 2.5%, *p* = 0.005); and first-year mortality was 8.3 times higher (5.0% vs. 0.6%, *p* = 0.006). The Charlson Comorbidity Index (CCI) and admission during the COVID-19 period were strong predictors of mortality, while the number of complications, age, and gender did not significantly influence the mortality rate. An increase of one point in CCI resulted in a 42% increase in the likelihood of mortality, while hospitalization during the COVID-19 period was associated with an odds ratio of 2.44 for death compared to the pre-COVID-19 period (*p* = 0.013, 95% CI [1.19, 4.94]). *Conclusions*: The excess mortality may be attributed to the COVID-19 pandemic because the groups were comparable in all other aspects (Barthel index, CCI, complications, age, and gender). Additional five-year mortality data will be useful for analyzing mortality dynamics, as pre-COVID-19 patients will enter the COVID-19 period and COVID-19 patients will enter the post-COVID-19 period.

## 1. Introduction

The onset of COVID-19 in Croatia is considered to be March 2020, when several positive cases were recorded, coinciding with the World Health Organization’s declaration of the COVID-19 pandemic [1]. Many hospitals reduced the number of operating rooms they had available and reorganized medical staff in response to patients infected with COVID-19 [2,3,4]. The COVID-19 pandemic led to a lockdown in Croatia, a ban on elective surgeries, a recommendation to shorten patients’ hospital stays, and the redirection of some health personnel to COVID-19 departments while maintaining an emergency program. This resulted in reduced capacity for surgery and non-COVID-19-patient care [5]. The COVID-19 pandemic caused 7.1 million deaths worldwide by Jan 2026 and has resulted in a decline in global life expectancy, as reported by the World Health Organization [6]. The estimated excess mortality is even higher, ranging from 19.1 million to 35.6 million worldwide. Cumulative mortality during the COVID-19 era in Croatia was reported to range from 14,963 to 18,803 [7,8]. Excess mortality was also observed in Croatia during the COVID-19 years, with the highest death rate in 2021 (16.2/1000 people), which can be compared to 12.9/1000 in pre-COVID-19 2018. [6]. Patients with hip fractures represent a highly vulnerable population with multiple comorbidities, and these fractures are a common source of future mortality and morbidity [9,10,11,12]. Interestingly, some studies have shown that the number of hip fractures reported decreased during the COVID-19 pandemic, with no difference in post-operative protocols, complications, or the type of hip fracture surgery employed [13,14,15,16], including lower or no difference in 30- or 90-day mortality [13,15,17,18,19]. On the other hand, other studies have shown that surgical treatment for fragility hip fractures during the COVID-19 pandemic resulted in worse short-term post-operative functional outcomes, higher complication rates compared to the pre-pandemic period, and higher 30-day, 6-month, and 1-year mortality [20,21,22]. Lower functional recovery may be related to the unavailability of physical therapy during the COVID-19 period [21,22,23]. These articles have found a correlation between short-term mortality after hip fracture surgery and the COVID-19 era, with a gap in knowledge regarding long-term mortality. The objective of this study was to analyze up to three-year mortality after surgical treatment of acetabular or femoral fractures among patients from the COVID-19 and pre-COVID-19 periods who underwent physical therapy in our rehabilitation ward. To ensure comparability, we included only patients who underwent 10-day acute inpatient rehabilitation in the rehabilitation ward. Additionally, we examined the association between mortality and patient comorbidity status, measured using the Charlson Comorbidity Index, as well as the number of complications during the hospital stay.

## 2. Materials and Methods

### 2.1. Study Design

This retrospective observational study was approved by the Ethical Committee of University Hospital Split (protocol number 500-03/22-01/197, 2022). Data were obtained from medical records from January 2018 to April 2022 from the Division of Physical Medicine and Rehabilitation with Rheumatology, University Hospital Split, Croatia. Patients were then coded, and the data were analyzed by other co-authors. The Ethical Committee waived patient consent on the condition that all patient data would be fully anonymized, with identities known only to the principal investigator. The study was reported in accordance with the Strengthening the Reporting of Observational Studies in Epidemiology (STROBE) guidelines.

### 2.2. Participants

Enrolled subjects met the following inclusion criteria: they were patients who had surgically treated acetabular or femoral fractures (hip fractures) and had been admitted to in-hospital physical therapy as a direct transfer from the surgical ward or from home, with a maximum of 14 days from the operation.

Baseline demographic and anthropometric data, as well as the condition leading to hospitalization, were recorded. The type of hip or femur fracture, type of operation, time from fracture to operation, total hospital stays (including both surgical and rehabilitation wards), mechanism of injury (fragility fall or otherwise), and corticosteroid use were also documented for both groups (pre-COVID-19 and COVID-19).

**Figure 1:**
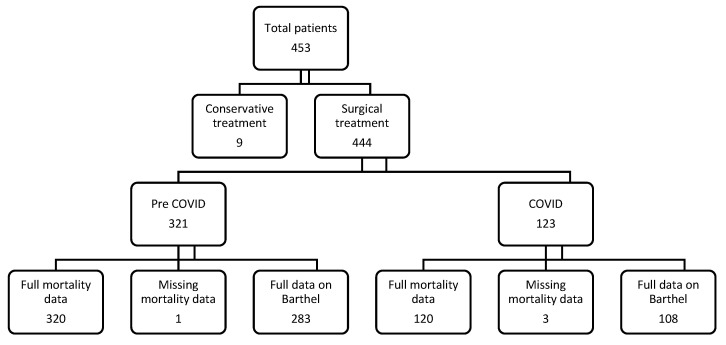
Chart of patients with operated-on acetabular and femoral fractures treated at our acute rehabilitation ward from January 2018 to April 2022.

Patients with acetabular and femoral fractures treated conservatively were excluded from the study. Patients with distal femoral fractures that had been operated on were also excluded from the study. In Figure 1, the total number of patients included in the study is shown. Mortality data were unavailable for one person in the pre-COVID-19 group and three people in the COVID-19 group. Missing Barthel data was found in 38 cases in the pre-COVID-19 group and 15 cases in the COVID-19 group. The unit of analysis was defined as the individual patient rather than fracture episode since there were no bilateral or repeated fractures in any of the cases analyzed. Nine patients were excluded because of conservative femoral neck fractures. Types of fractures and operations are presented in Table 1.

### 2.3. Data Measurement

The Barthel Index, which measures an individual’s functional independence in activities of daily living (ADLs), was assessed twice by experienced physiotherapists: at admission and at discharge as a part of routine assessment. A Barthel index score of up to 60 represents severe loss of mobility and self-care independence, while a score of 100 corresponds to an independent patient. The Charlson Comorbidity Index (CCI) was calculated at admission. All medical complications during the ten-day hospitalization in the rehabilitation department were recorded. Physical therapy was done by experienced physiotherapists according to the post-operative protocols for femoral osteosyntheses or hip prosthesis, including passive and active exercises for hip and thigh muscles, respiratory exercises, assistance in transfer (into a bed and from the bed to a chair), verticalization, walking with an assistive device twice a day, walking on stairs, and education on restrictions due to hip prosthesis. Physical therapy did not differ during the COVID-19 period in terms of the number of physiotherapists or the duration of physical therapy. The thromboprophylaxis regimen was active in view of recent hip surgery. Each patient’s mandatory health insurance status was checked annually in state registries, and the date of death was recorded if insurance was terminated.

### 2.4. Outcomes

The primary outcome of this study consisted of yearly and cumulative 3-year mortality among patients with surgically treated acetabular or femoral fractures who underwent 10 days of in-hospital rehabilitation, comparing the pre-COVID-19 period with the COVID-19 period.

### 2.5. Secondary Outcome Measures

We compared the levels of activities of daily living (ADLs) in the pre-COVID-19 and COVID-19 groups upon discharge from the hospital as measured via the Barthel index.

We analyzed the association between mortality rate and increased comorbidity upon admission in the pre-COVID-19 and COVID-19 eras using CCI.

We analyzed the association between mortality rate and the number of complications during the hospital stay in the pre-COVID-19 and COVID-19 eras, using data from medical records to count complications during hospital stays.

### 2.6. Statistical Analysis

Analyses were conducted in R (R Core Team, 2023, Vienna, Austria). The level of statistical significance was set at α = 0.05 (*p* < 0.05). Descriptive statistics were reported for all variables. Data for age, time in hospital, time until surgery, and Barthel score were presented as means ± standard deviations. The normality of continuous variables was assessed using the Shapiro–Wilk test. As the Shapiro–Wilk test indicated there was a non-normal distribution of continuous variables, the Kruskal–Wallis rank sum test was used to compare continuous variables between the COVID-19 and pre-COVID-19 groups. Categorical variables were compared using either Fisher’s exact test or Pearson’s chi-squared test, as appropriate. Differences in Barthel index scores over time were examined using the Wilcoxon signed-rank test. Additionally, the effects of time point, number of comorbidities, and COVID-19 period group on these scores were evaluated using a linear mixed-effects model, allowing a comprehensive analysis of the factors influencing Barthel index variations. Multicollinearity among predictors was assessed using variance inflation factors (VIFs).

## 3. Results

Descriptive data from patients in the pre-COVID-19 and COVID-19 groups are presented in Table 2.

There was a 38% decrease in the number of patients in the COVID-19 group, while the number of patients hospitalized for the diagnosis, classified as S72.0, S72.1, S72.2, and S72.3, remained constant over the years analyzed (555—2018; 569—2019; 516—2020; 651—2021; and 588—2022).

Both groups consisted of patients enrolled over the same 26-month period. The initial or final Barthel score was most frequently missing (Figure 1). Data used for mortality analysis, found in the obligatory medical insurance database, were missing for individuals who moved to another country.

The ages of the patients in the groups did not differ (*p* = 0.989). A female predominance was observed, with ratios of 2.52:1 in the pre-COVID-19 group and 2.72:1 in the COVID-19 group. The time from injury to operation and the length of combined surgical and rehabilitation hospital stays were comparable between the pre-COVID-19 and COVID-19 groups. Most patients in both groups spent 8 to 13 days in the rehabilitation ward. Three out of four patients were admitted as direct transfers from the surgical ward, while 25% were admitted after a short period of out-of-hospital recovery (due to a lack of capacity for direct transfer or delays caused by antibiotic treatment).

The falls that led to fractures could be classified as either low- or high-impact, resulting in fragility fractures in more than 80% in both groups.

The most common reason for hospitalization was proximal femoral fracture surgery, accounting for 93% of cases in the pre-COVID-19 group and 85% in the COVID-19 group (Table 1).

The Barthel index showed that the majority of both groups were admitted with severe dependency (43.84 ± 22.32 pre-COVID-19 vs. 45.41 ± 23.01 during COVID-19, *p* = 0.979). Both groups recovered to a point of mostly moderate dependency, with no differences between the groups (65.2 ± 22.65 pre-COVID-19 vs. 64.9 ± 23.69 during COVID-19, *p* = 0.99).

**Figure 2:**
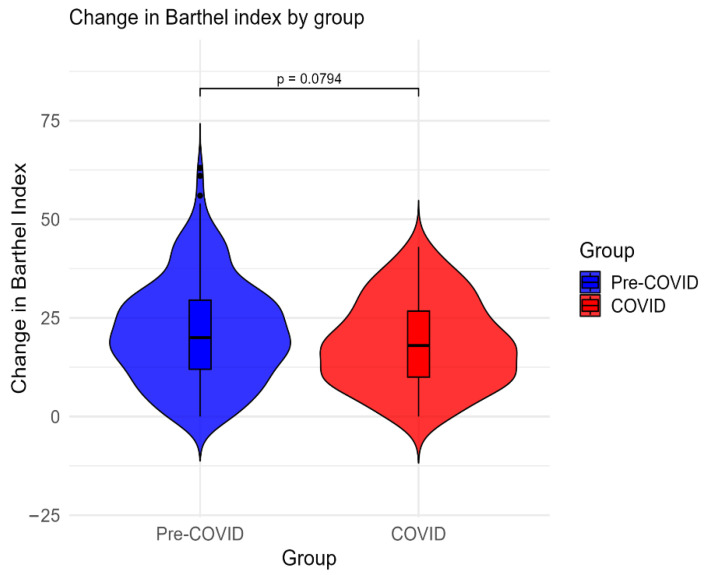
Change in Barthel index scores in the pre-COVID-19 and COVID-19 groups.

The efficacy of physical therapy was analyzed using the change in Barthel index scores from admission to discharge. Both the pre-COVID-19 group (W = 36,046, *p* < 0.001, r = 0.86) and the COVID-19 group (W = 4560, *p* < 0.001, r = 0.87) showed large improvements in Barthel index scores from admission to discharge, with no significant difference in the change in Barthel index between groups (W = 15,515, *p* = 0.079), as shown in Figure 2.

The quantities of complications during hospitalization were similar between the groups: 57.3%, 30.8%, 9%, and 2.8% for zero, one, two, or three or more complications in the pre-COVID-19 group, and 54.4%, 31.7%, 10.6%, and 3.3% in the COVID-19 group, respectively.

During the COVID-19 period, there was a statistically higher incidence of deep venous thrombosis than in the pre-COVID-19 period (confirmed by lower-limb Doppler ultrasound), as shown in Table 3.

The CCI showed that the groups did not differ in terms of the number or severity of comorbidities at admission (3.67 ± 1.89 pre-COVID-19 vs. 3.98 ± 1.92 during COVID-19, *p* = 0.434).

The total three-year cumulative mortality for both groups was 8.4% (n = 37), with higher mortality in the COVID-19 group (14.2%, n = 17) than in the pre-COVID-19 group (6.2%, n = 20) (χ^2^ = 6.16; df = 1; *p* = 0.013). Fisher’s exact test showed the most significant difference in first-year mortality between the pre-COVID-19 group (0.6%, n = 2) and the COVID-19 group (5.0%, n = 6; *p* = 0.006). The odds of death were 8.35 times higher in the COVID-19 group (95% CI [1.47, 85.87]). Pearson’s chi-square test with Yates’ continuity correction indicated a significant difference in two-year mortality between the groups (χ^2^ = 7.89; df = 1; *p* = 0.005), with higher mortality in the COVID-19 group (9.2%, n = 11) than in the pre-COVID-19 group (2.5%, n = 8). The number of deaths during the follow-up period is presented in Table 4.

**Table 4:** Cumulative deaths in the 1st, 2nd, and 3rd year in pre-COVID-19 and COVID-19 periods.

Yearly Mortality (n)	1st	2nd	3rd	All Patients (n)
Pre-COVID-19	2 (0.6%)	8 (2.5%)	20 (6.2%)	320
COVID-19	6 (5.0%)	11 (9.2%)	17 (14.2%)	120
Total	8 (1.8%)	19 (4.3%)	37 (8.4%)	440

**Figure 3:**
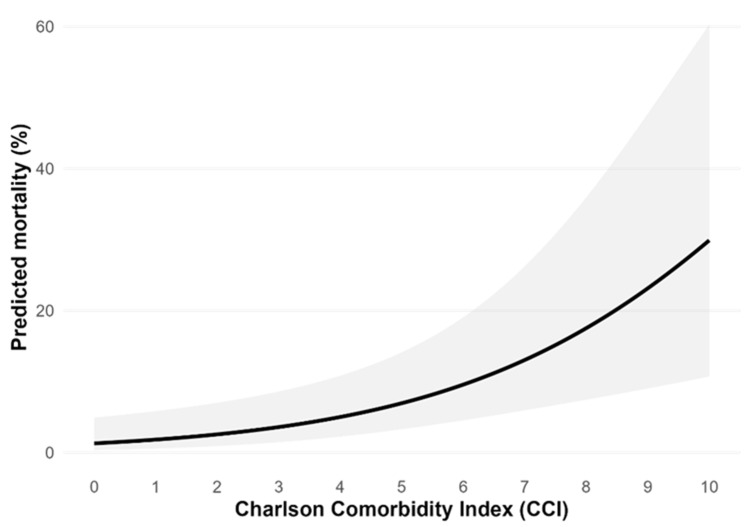
Charlson Comorbidity Index scores and mortality in the logistic regression model among patients with surgically treated hip and femoral fractures.

A logistic regression was conducted to examine whether the number of complications, the CCI, group allocation (pre-COVID-19 vs. COVID-19), age, and sex predicted mortality. The analysis included 433 complete cases; eight observations were excluded due to missing data for at least one predictor. No evidence of multicollinearity was observed (all VIFs ≤ 1.11). The model demonstrated modest explanatory power (Nagelkerke R^2^ = 0.14) and a significantly improved fit over the null model (likelihood ratio χ^2^ = 26.80, *p* < 0.001). The Hosmer–Lemeshow goodness-of-fit test indicated no evidence of a poor fit (χ^2^ = 7.995, df = 8, *p* = 0.434). CCI scores were positively associated with mortality, with higher CCI scores linked to greater odds of death (β = 0.35, *p* = 0.001, OR = 1.42, 95% CI [1.14, 1.76]). Each one-unit increase in the CCI resulted in a 42% increase in the likelihood of mortality. Group allocation was also significantly associated with mortality, with patients admitted during the COVID-19 period having more than twice the odds of death compared to those admitted during the pre-COVID-19 period (β = 0.89, *p* = 0.013, OR = 2.44, 95% CI [1.19, 4.94]). In contrast, the number of complications (β = −0.12, *p* = 0.594, OR = 0.89, 95% CI [0.56, 1.34]), age (β = 0.03, *p* = 0.113, OR = 1.03, 95% CI [1.00, 1.07]), and sex (β = 0.04, *p* = 0.923, OR = 1.04, 95% CI [0.46, 2.60]) were not significantly associated with mortality. Overall, the model in our study indicated that the CCI and admission during the COVID-19 period were predictors of mortality (Figure 3), while the number of complications, age, and sex did not significantly influence mortality.

## 4. Discussion

This study aimed to investigate whether there was an increase in long-term mortality among patients with surgically treated hip or femoral fractures who underwent acute rehabilitation in the pre-COVID-19 and COVID-19 periods. These patients, aged 18 and older, had a combined cumulative three-year mortality rate of 8.4% (including both the COVID-19 and pre-COVID-19 periods). When analyzed separately, patients from the COVID-19 period had greater mortality in all three years, with the greatest difference observed in the first year, showing 8.3-fold more deaths. In the second and third years, cumulative mortality gradually decreased, with 3.7- and 2.3-fold more deaths, respectively. All deaths in our study occurred outside the hospital, and there were no COVID-19-positive patients in our rehabilitation department during the observed period. The high mortality rate for COVID-19 infection and low vaccination rates throughout Croatia during the pandemic could have partly influenced the higher mortality after discharge [24]. In previous studies, some significant factors other than COVID-19 independently influenced mortality after a surgically repaired hip fracture: older age, male gender, American Society of Anesthesiologists (ASA) score ≥ 3, CCI score ≥ 3, delay in operation > 48 h, complications during hospitalization, long post-operative hospital stay, frailty, poor ambulation before fracture, non-operative fracture management, congestive heart failure, dementia or cognitive impairment, Parkinson’s disease, malignancy, and peri-operative hypotension [9,25,26,27].

Our groups were compatible in terms of gender, age, time until operation, type of fracture, percentage of fragility falls, number of complications during hospitalization, post-operative stay, Barthel index, and CCI scores.

Previous studies on 3-year mortality after hip fracture, from before the COVID-19 pandemic, showed an increase in mortality from 14% in the first year to 40% in the third year among elderly patients [9,12]. As far as we know, no data on cumulative three-year mortality after hip or femoral fracture during the pandemic are available. In the literature, short-term mortality rates after hip fracture during the COVID-19 pandemic vary greatly. Thirty-day mortality after a geriatric hip fracture during the COVID-19 pandemic ranged from 2.4% to 14% in COVID-19-negative to 38% in COVID-19-positive patients [22,28], while mortality in the first year ranged from 4% to 43% in COVID-19-negative to 54.5% in COVID-19-positive patients [18,22]. Factors like lack of professional personnel due to COVID-19 ward relocation, lack of fracture liaison service implementation for optimal treatment of osteoporotic fragility hip fractures, and inadequate rehabilitation could influence short-term excess mortality, with early ambulation, functional mobility, and intensive hospital rehabilitation serving as protective factors [18,25]. Some studies have shown low or unchanged short-term mortality after a hip fracture during the COVID-19 period and tried to explain this via the reduced number of hip fracture patients, optimal preparation for a pandemic, infection control measure implementation, prioritization of high-risk patients, and proper coordination between healthcare providers. Also, the fact that older people engage in fewer outings and self-isolation due to quarantine lead to a decrease in the total number of patients, which could increase the emergency capacity for non-COVID-19-patient treatment, including for hip fracture surgery [15,17,18].

In our study, a 38% decrease in admission to the rehabilitation ward for hip and femoral fractures during the COVID-19 period was observed. There were no significant differences in total orthopedic trauma patient admission between the groups, with 1228 fractures in the pre-COVID-19 group and 1248 in the COVID-19 group. The observed decrease in the number of patients in our department can be explained by the reorganization of the department, with a reduction in the number of available beds, which is in line with other studies [23,29]. However, despite the reduced number of rehabilitated patients, functional outcomes between the observed groups were the same, indicating comparable levels of rehabilitation. Increased long-term mortality was observed in the COVID-19 pandemic despite the comparability of the groups. The main predictors of mortality in our study were the Charlson Comorbidity Index (CCI) and the COVID-19 period. Each additional point in the CCI increased the probability of mortality by 42%, while the COVID-19 period itself resulted in a 2.44 odds ratio of mortality compared to the pre-COVID-19 period. Perhaps not only COVID-19 as a disease but also certain factors like reduced social support, delayed outpatient follow-up, or post-discharge care disruptions could influence the treatment of other diseases and contribute to higher long-term mortality [20,22,30]. In Croatia and Split, the mortality of bedridden geriatric patients with operated-on femoral neck fractures was studied previously and found to be 15% in the first year after discharge from the rehabilitation department [31]. However, both pre-COVID-19 (0.8%) and COVID-19 (5%) one-year mortality rates are well below these high numbers. Although the majority of patients were over 70 years of age, most were not bedridden. Bedridden and immobile patients are very sensitive to complications; those with zero complications had a 3% mortality rate, while among those with three or more complications, the mortality rate surged to a dramatic 61% in the first year [31]. In contrast to the cited article, which shows a correlation between mortality and complications in geriatric inpatients, we did not observe this connection, possibly because our cohort selection process was different. This study included patients who were mobile at the time of admission (using mobility aids and under physiotherapist’s supervision), as shown by the Barthel index score upon admission (32 vs. 45 out of 100). Our study found a correlation between the comorbidity status of a patient and three-year survival, which is in accordance with Aguado et al.’s research [25].

Other articles have found higher mortality during the COVID-19 pandemic in disease categories like ischemic stroke and neoplasms, which could highlight the pandemic’s impact, leading to a decrease in life expectancy [32,33]. Five-year follow-ups on mortality are planned for this cohort to confirm whether this excess mortality is purely because of the COVID-19 pandemic. By that time, the pre-COVID-19 group will have entered the COVID-19 period, and the COVID-19 group will have entered the post-COVID-19 period. This is partly demonstrated in this paper by the gradual leveling of cumulative mortality from 8.3× to 3.7× and 2.3× for the first, second, and third years as the pre-COVID-19 group entered COVID-19 era. Future research should incorporate time-to-event analytical approaches, such as the Cox proportional hazards model, to better capture the temporal dynamics of mortality outcomes.

### Limitations of This Study

This monocentric study examined only a subgroup of hip and femoral fracture patients who had been operated on. The patients analyzed had enough locomotor capacity to undergo intensive physical therapy but could not undergo ambulatory physical therapy. Patients with lower capacity were usually treated with home-based physiotherapy and were not included in mortality data. This limited the value of general population mortality after an operated hip/femoral fracture. Due to the retrospective nature of this study intra- and inter-observer variability, the Barthel score could not be determined between physiotherapists. This study cannot show the exact causes of mortality because the death data were collected through administrative insurance data. There was a complete lack of socioeconomic and post-discharge care variables that could be of interest in analyzing long-term survival, along with a gap in the percentage of unknown vaccination status.

## 5. Conclusions

The COVID-19 pandemic was a valuable lesson for humanity regarding the organizational requirements of health systems. All hospital departments must be prepared for pandemics by maintaining accessibility during public health emergencies. The 38% drop in operated-on hip fracture patients admitted to our hospital’s rehabilitation ward is big, although the quality of physical therapy was preserved. Our study helps reveal the factors that influenced three-year mortality during the COVID-19 pandemic, which were the Charlson Comorbidity Index and the COVID-19 period itself. An increase of one point in the CCI resulted in a 42% increase in the likelihood of mortality, while hospitalization during the COVID-19 period was associated with an odds ratio of 2.44 for death compared to the pre-COVID-19 period. Patients with a high comorbidity status are at the greatest risk of death in the COVID-19 period and should be protected from delayed outpatient follow-ups for both post-operative conditions and other chronic-disease check-ups.

## Figures and Tables

**Table 1:** Types of fractures and surgeries.

		Pre-COVID-19	COVID-19
Fracture types	Femoral neck	167 (52.8%)	48 (39%)
	Trochanteric	124 (39.2%)	56 (45.5%)
	Acetabular	9 (2.8%)	5 (4.1%)
	Shaft and periprosthetic	16 (5.1%)	14 (11.4%)
Type of operation	Hip replacement	161 (50.9%)	44 (35.8%)
	Intramedullary rod placement	132 (41.8%)	62 (50.4%)
	Application of metal plate and screws	21 (6.6%)	17 (13.8%)
	External fixation	2 (0.6%)	0 (0%)

**Table 2:** Demographics and clinical data pertaining to the patients with femoral and acetabular fractures.

Group		Pre-COVID-19	COVID-19
Patients in trauma ward		1228	1248
Patients in rehab ward		321	123
Age		72.78 ± 15.53	73.21 ± 17.93
Gender	Male	91 (28.3%)	33 (26.8%)
	Female	230 (71.7%)	90 (73.2%)
Time from fracture to operation (days)		2.8 ± 2.6	1.98 ± 2.14
Total hospitalization (days)		19.33 ± 6.75	19.39 ± 7.25
Rehabilitation ward stay (days)		10.5 ± 2.39	11.63 ± 3.58
Transfer from surgery ward	Yes	237 (73.8%)	92 (75.4%)
	No	84 (26.2%)	30 (24.6%)
Fragility fall	Yes	271 (84.7%)	100 (81.3%)
	No	49 (15.3%)	23 (18.7%)
Corticosteroid use	Yes	9 (2.8%)	5 (4.1%)
	No	312 (97.2%)	118 (95.9%)
Previous COVID-19 infection	Yes	-	15 (12.2%)
	No	-	35 (28.5%)
	Unknown	-	73 (59.3%)
Vaccination status	Yes	-	40 (32.5%)
	No	-	22 (17.9%)
	Unknown	-	61 (49.6%)

**Table 3:** Type and number of post-operative and acute rehabilitation ward stay complications.

Complications	Pre-COVID	COVID	*p*
Urinary infection	106	48	0.27
Urinary retention	14	6	0.8
Delirium	12	5	1
Decubital ulcer	20	6	0.66
Infection	6	2	0.679
Peripheral nerve injury	3	1	1
Deep venous thrombosis	0	4	0.007
Pulmonary embolus	4	1	1
Pneumonia	2	2	0.584
Transfusion due to anemia	4	4	0.239
Heart decompensation	6	2	1
Hip dislocation	6	1	0.68
Other	6	2	1

## Data Availability

The raw data supporting the conclusions of this article will be made available by the authors on request.
